# Body composition and risk of gastric cancer: A population‐based prospective cohort study

**DOI:** 10.1002/cam4.3808

**Published:** 2021-02-23

**Authors:** An‐Ran Liu, Qiang‐Sheng He, Wen‐Hui Wu, Jian‐Liang Du, Zi‐Chong Kuo, Bin Xia, Yan Tang, Peng Yun, Eddie C. Cheung, You‐Zhen Tang, Yu‐Long He, Chang‐Hua Zhang, Jin‐Qiu Yuan, Gang Sun

**Affiliations:** ^1^ Department of Clinical Nutrition The Seventh Affiliated Hospital Sun Yat‐sen University Guangdong Province China; ^2^ Clinical Research Center Big Data Center The Seventh Affiliated Hospital Sun Yat‐sen University Guangdong Province China; ^3^ Center for Digestive Disease The Seventh Affiliated Hospital Sun Yat‐sen University Guangdong Province China; ^4^ Division of Medical Record Management The Seventh Affiliated Hospital Sun Yat‐sen University Guangdong Province China; ^5^ Department of Endocrinology The Seventh Affiliated Hospital Sun Yat‐sen University Guangdong Province China; ^6^ Division of Gastroenterology School of Medicine University of California Davis Davis CA USA

**Keywords:** cohort study, fat mass, fat‐free mass, gastric cancer, UK Biobank

## Abstract

The recognition of adiposity as a risk factor for gastric cancer is mainly based on traditional anthropometric indices, such as body mass index, which are unable to discriminate between lean and fat mass. We undertook this study to examine body composition and subsequent risk of gastric cancer. This is a prospective analysis of participants free of cancer from the UK Biobank. We measured baseline body composition with electrical bioimpedance analysis and confirmed cancer diagnosis through linkage to cancer and death registries. We evaluated hazard ratios (HRs) and confidence interval (CIs) with COX models adjusting for potential confounders. We documented 326 cases of cancer from 474,929 participants over a median follow‐up of 6.6 years. Both male (HR 1.70, 95% CI 1.01 to 2.89) and female participants (HR 2.47, 95% CI 1.15 to 5.32) in the highest quartile of whole body fat‐free mass were associated with increased risk of gastric cancer as compared with those in the lowest quartile.Whole body fat mass was associated with a decreased risk of gastric cancer (HR per 5‐unit increase 0.86, 95% CI 0.74 to 0.99) in females, but not in males. We concluded that fat‐free mass and fat mass may have different effects on gastric cancer risk. This study provided evidence for individualized weight management for the prevention of gastric cancer.

## Graphical abstract

Based on UK biobank cohort (474,929 participants and 326 cases of gastric cancer), this study found fat‐free mass is assocated with increased risk of gastric cancer in both genders while fat mass is associated with reduced risk of gastric cancer in females. Our results provide new insights into relationships between obesity and gastric cancer, and deliver important clinical and public health messages about healthy body composition beyond BMI.

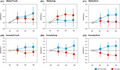

## 1 INTRODUCTION

Gastric cancer is one of the most common and deadly cancers worldwide.^1^, ^2^ Data from the GLOBOCAN database showed that gastric cancer was responsible for over 1,033,000 new cases and 78,200 deaths in 2018, ranking fifth for cancer incidence and third for cancer mortality.^1^ The global incidence of gastric cancer has declined rapidly over the last few decades, possibly due to the recognition of modifiable risk factors such as *H. pylori* infection, diet, and smoking.^2^, ^3^ Overweight and obesity have been considered a risk factor for many types of cancer. A prospective study of more than 900,000 U.S. adults indicated that obesity caused approximately 20% of cancer deaths in women and 14% in men, making obesity the second biggest preventable cause of cancer.^4^ For gastric cancer, epidemiological studies investigating the contribution of excess body weight are increasing, but findings were inconsistent. In 2014, a meta‐analysis showed that obesity was associated with an increased risk of gastric cancer,^5^ while a recent meta‐analysis suggested overweight might be a protective factor in gastric cancer risk of Asian adults.^6^

For most previous studies evaluating obesity and gastric cancer risk, traditional anthropometric measures, including body mass index (BMI) and waist circumference (WC), were used as the exposure measurements. Although these indices provide simple, cheap, and crude measures of body size, they could neither directly discriminate between lean and fat mass, nor precisely evaluate the distribution of fat mass. It has been shown that lower body, upper body, and visceral fat deposits have unique characteristics with regards to fatty acid metabolism.^7^ Epidemiological evidence concerning the association between body composition and gastric cancer risk has emerged but is still inadequate. A cohort study of 41,295 participants found that nonfat component of weight was associated with increased risk of gastric cardia adenocarcinoma.^8^ Due to its small number of case (n = 98), inadequate adjustment for important confounders such as diet, and lack of assessment for non‐linear association, further studies are still required. Further investigation of the association between body composition and gastric cancer could (1) identify the effects of key composition, fat, or lean mass, on gastric cancer development, and (2) assess the distribution of fat or lean mass and risk of gastric cancer. These results may provide evidence for individualized weight management for the prevention of gastric cancer.

UK Biobank is a large prospective study which collected data about body composition and cancer incidence from 0.5 million UK adults. Based on the UK Biobank dataset, we carried out this prospective analysis to confirm the relationship between body composition (including total/trunk/arm/leg body fat mass and body fat‐free mass) and risk of gastric cancer.

## 2 METHODS

### 2.1 Study design and data source

This is a population‐based prospective cohort study based on the UK Biobank dataset (reference number 51671, May 2019). At recruitment in 2006–2010, the participants underwent a range of physical measurements and detailed assessments of health‐related factors. Blood, urine, and saliva samples were also collected for biochemical analysis. Follow‐up was conducted through linkages to routinely available national datasets. Details of the rationale, design and survey methods for UK Biobank can be found elsewhere.^9^ For the present analysis, we included all participants from UK Biobank who had complete data for fat‐free mass and fat mass. We excluded the participants with a diagnosis of cancer prior to baseline assessment (except for non‐melanoma skin cancer ICD‐10 C44). Finally, we included 465,292 participants (see the flowchart of study selection in *Electronic supplementary material* Figure S2). UK Biobank was approved by the North West Multi‐centre Research Ethics Committee (MREC), the Patient Information Advisory Group (PIAG) in England and Wales, and the Community Health Index Advisory Group in Scotland (CHIAG).

### 2.2 Body composition and anthropometry

UK Biobank evaluated baseline fat free mass (kg) and fat mass (kg) with electrical bio‐impedance analysis (Tanita BC418MA body composition analyser) of over 93% participants. Fat mass is the actual weight of fat in body. Fat mass is the actual weight of fat in body. Fat free mass is all body components (excluding fat), including internal organs, skeletal muscle, bone, and body water. (*Electronic supplementary material* Figure S1) The whole body as well as site‐specific (trunk, leg, arm) fat‐free mass/fat mass were evaluated. Detailed descriptions of the procedures used to measure body composition is available on the study website.^10^ UK Biobank also evaluated body composition in 5,170 participants using dual‐energy X‐ray absorptiometry (DXA). Assessment of body composition by bio‐impedance and DXA showed high correlation (fat‐free mass: r = 0.96, fat mass: r = 0.86). Trained staff measured standing height using the Seca 202 device (Seca, Hamburg, Germany) and assessed waist/hip circumference with the Wessex non‐stretchable sprung tape measure (Wessex, United Kingdom). BMI was calculated by dividing body weight (in kilograms) by height squared (in meters squared).

### 2.3 Ascertainment of cancer cases

UK Biobank obtains data on cancer diagnoses from the Health & Social Care Information Centre for participants in England and Wales, and the NHS Central Register for participants in Scotland. These registrations recorded diagnosis of cancer and cancer deaths using the 10th revisions of international classification of diseases (ICD‐10) codes. The primary outcome for this study was gastric cancer (C16). We also evaluated gastric cardia adenocarcinoma (GCA) and non‐gastric cardia adenocarcinoma (NGCA) in the secondary analyses.

### 2.4 Data analysis

We calculated person‐years from the recruitment date to the date of the first diagnosis of cancer, death, or the last date of follow‐up (30 October 2015), whichever came first. The analyses were carried out separately in males and females due to effect modification. We evaluated the risk of gastric cancer by quartiles and as continuous per 5‐unit increase in fat‐free mass/fat mass. To compare the ability of predicting gastric cancer across various body composition and anthropometry measures, we evaluated the HRs per standard deviation (SD) increase with Cox regression. To investigate potential nonlinear associations of fat free mass / fat mass with gastric cancer risk, we fitted restricted cubic splines with four knots at the 5th, 35th, 65th, and 95th percentiles in Cox models. We checked the proportional hazards assumption using Schoenfeld's tests. For covariates with selections of ‘do not know’ and ‘prefer not to answer’, or with missing covariate data, we included an “unknown/missing” indicator.

Because the effect of body composition on gastric cancer shows a gender difference, the analyses were stratified by gender. To control potential confounding effects, we stratified the analyses by age in the basic Cox regression model. Additionally, we adjusted for ethnic, index of multiple deprivation, alcohol consumption, smoking status, physical activity, fruit and vegetable intake, diabetes, Nonsteroidal anti‐inflammatory drugs (NSAIDs) use, height, and family history of cancer in the multivariate Cox regression. For the analysis of body composition, we included both fat‐free mass and fat mass in the model to examine their independent effects.

We performed a number of sensitivity analyses to check the robustness of the primary analysis. First, we limited the participants in people with a follow up time over 2 years. Second, we additionally adjusted for gastro‐oesophageal reflux/gastric reflux, gastric/stomach ulcers, gastritis/gastric erosions and proton‐pump inhibitors (PPIs) use. Third, we adjusted for hormone replacement therapy (HRT) and Optical Coherence Tomography (OCT) in females to investigate potential residual confounding effect. Forth, we excluded participants with self‐reported cancer diagnosed by doctor. Lastly, we additional adjusted for red meat consumption to investigate potential influence. Statistical analyses were conducted using the SAS (release 9.4; SAS Institute Inc) and R software (version 3.5.0, R Foundation for Statistical Computing, Vienna, Austria).

## 3 RESULTS

This study included a total of 465,292 participants, of which 213,843 were males and 251,449 were females (Table 1). For both males and females, age was likely to increase with whole body fat mass while decrease with whole body fat‐free mass. The participants with lower whole body fat mass or whole‐body fat‐free mass tended to do more physical activity and have a lower rate of hypertension and diabetes.

**TABLE 1:** Characteristics of study participants

	Whole body fat‐free mass, Quartile				Whole body fat mass, Quartile			
	Quartile 1	Quartile 2	Quartile 3	Quartile 4	Quartile 1	Quartile 2	Quartile 3	Quartile 4
Males								
N	53274	52897	54132	53540	53400	53301	53361	53781
Mean (SD) age, years	58.5 (7.89)	57.2 (8.10)	56.1 (8.13)	54.4 (8.09)	55.2 (8.38)	56.5 (8.26)	57.1 (8.09)	57.3 (7.87)
White, n (%)	47880 (89.9%)	49911 (94.4%)	51714 (95.5%)	51495 (96.2%)	49720 (93.1%)	49850 (93.5%)	50264 (94.2%)	51166 (95.1%)
Current smoker, n (%)	8059 (15.1%)	6246 (11.8%)	6178 (11.4%)	6199 (11.6%)	8061 (15.1%)	6394 (12.0%)	6126 (11.5%)	6101 (11.3%)
Alcohol intake (daily or almost daily), n (%)	14175 (26.6%)	14116 (26.7%)	13943 (25.8%)	11942 (22.3%)	13934 (26.1%)	14254 (26.7%)	13844 (25.9%)	12144 (22.6%)
Mean (SD) MET, minutes/week	2890 (3010)	2860 (2930)	2840 (2950)	2680 (2920)	3240 (3160)	2910 (2950)	2730 (2880)	2370 (2740)
Mean (SD) fruit and vegetable intake, portions/day	4.11 (3.27)	4.22 (3.12)	4.27 (3.11)	4.25 (3.03)	4.37 (3.34)	4.25 (3.18)	4.14 (3.04)	4.10 (2.97)
Mean (SD) BMI, kg/m2	24.9 (2.79)	26.7 (2.86)	28.3 (3.22)	31.5 (4.59)	23.7 (1.94)	26.3 (1.66)	28.4 (1.77)	33.0 (3.76)
Hypertension, n (%)	14139 (26.5%)	14735 (27.9%)	16136 (29.8%)	19055 (35.6%)	9241 (17.3%)	13363 (25.1%)	17265 (32.4%)	24196 (45.0%)
Diabetes, n (%)	2853 (5.4%)	2935 (5.5%)	3476 (6.4%)	5268 (9.8%)	1578 (3.0%)	2290 (4.3%)	3452 (6.5%)	7212 (13.4%)
Females								
N	62227	63144	62227	63851	62702	62761	62841	63145
Mean (SD) age, years	57.9 (7.67)	56.5 (7.93)	55.6 (8.05)	54.5 (8.02)	54.6 (8.19)	56.3 (7.99)	57.1 (7.84)	56.5 (7.81)
White, n (%)	57142 (91.8%)	60107 (95.2%)	59397 (95.5%)	60072 (94.1%)	59126 (94.3%)	59457 (94.7%)	59364 (94.5%)	58771 (93.1%)
Current smoker, n (%)	5886 (9.5%)	5508 (8.7%)	5266 (8.5%)	5777 (9.0%)	6423 (10.2%)	5392 (8.6%)	5373 (8.6%)	5249 (8.3%)
Alcohol intake (daily or almost daily), n (%)	10611 (17.1%)	11196 (17.7%)	10344 (16.6%)	8244 (12.9%)	12346 (19.7%)	11335 (18.1%)	9808 (15.6%)	6906 (10.9%)
Mean (SD) MET, minutes/week	2610 (2520)	2620 (2510)	2540 (2460)	2320 (2400)	2900 (2650)	2640 (2490)	2440 (2410)	2080 (2250)
Mean (SD) fruit and vegetable intake, portions/day	4.94 (3.18)	4.97 (3.00)	4.99 (2.98)	4.92 (3.09)	5.12 (3.24)	5.03 (3.02)	4.93 (3.00)	4.74 (2.97)
Mean (SD) BMI, kg/m2	23.7 (3.01)	25.4 (3.33)	27.2 (3.83)	31.9 (5.82)	22.0 (1.82)	24.9 (1.70)	27.7 (2.02)	33.8 (4.58)
Hypertension, n (%)	12681 (20.4%)	12978 (20.6%)	14110 (22.7%)	20081 (31.4%)	8087 (12.9%)	11928 (19.0%)	16048 (25.5%)	23787 (37.7%)
Diabetes, n (%)	1341 (2.2%)	1422 (2.3%)	1983 (3.2%)	4518 (7.1%)	850 (1.4%)	1316 (2.1%)	2016 (3.2%)	5082 (8.0%)

SD, standard deviation; MET, Metabolic Equivalent for Task; BMI, body mass index.

We documented 326 cases of gastric cancer over a median follow‐up of 6.6 years. Table 2 presents the associations of whole‐body fat‐free mass/whole body fat mass and the risk of gastric cancer. For males, those in the highest quartile of whole‐body fat‐free mass had a 70% increased risk of gastric cancer as compared with those in the lowest quartile (Adjusted HR 1.70, 95% CI 1.01 to 2.89). For female participants, there was a similar trend that whole body fat‐free mass was associated with an increased risk of gastric cancer (*P*‐*trend* = 0.02). Compared with females in the lowest quartile of whole‐body fat‐free mass, those in the highest quartile was associated with a 2.47 times greater risk of gastric cancer (Adjusted HR 2.47, 95% CI 1.15 to 5.32). The associations between whole body fat mass and gastric cancer were likely to vary between males and females. For males, we did not observe sufficient evidence of an association. While in female participants, whole body fat mass was associated with a decreased risk of gastric cancer (Adjusted HR per 5‐unit increase 0.86, 95% CI 0.74 to 0.99). We evaluated the non‐linear associations between various anthropometric measurements with gastric cancer, and none showed sufficient evidence of non‐linearity (*Electronic supplementary material* Figure S3).

**TABLE 2:** Associations between whole body fat free mass/whole body fat mass and risk of gastric cancer

	Males				Females			
	Case/person‐years	Hazard Ratio (95% Confidence Interval)			Case/person‐years	Hazard Ratio (95% Confidence Interval)		
		Model 1	Model 2	Model 3		Model 1	Model 2	Model 3
Whole body fat‐free mass Quartiles								
Quartile 1	46/346049	1.00(Reference)	1.00(Reference)	1.00(Reference)	23/410209	1.00(Reference)	1.00(Reference)	1.00(Reference)
Quartile 2	67/345128	1.62(1.11, 2.36)^a^	1.72(1.16, 2.56)^b^	1.64(1.09, 2.46)^a^	22/416519	1.05(0.59, 1.89)	1.07(0.59, 1.96)	1.26(0.68, 2.34)
Quartile 3	58/353914	1.49(1.01, 2.20)^a^	1.60(1.04, 2.45)^a^	1.49(0.94, 2.35)	24/409769	1.26(0.71, 2.23)	1.28(0.70, 2.35)	1.73(0.89, 3.36)
Quartile 4	58/349725	1.76(1.19, 2.61)^b^	1.86(1.17, 2.96)^b^	1.70(1.01, 2.89)^a^	28/419038	1.59(0.91, 2.77)	1.52(0.81, 2.84)	2.47(1.15, 5.32)^a^
P‐trend		0.01	0.02	0.12		0.08	0.16	0.02
Continuous per 5‐unit increase Whole body fat mass Quartiles		1.11(1.02, 1.21)^a^	1.11(1.00, 1.23)	1.09(0.95, 1.24)		1.23(1.02, 1.49)^a^	1.21(0.98, 1.50)	1.52(1.13, 2.03)^b^
Quartile 1	37/349876	1.00(Reference)	1.00(Reference)	1.00(Reference)	23/414586	1.00(Reference)	1.00(Reference)	1.00(Reference)
Quartile 2	55/348536	1.36(0.89, 2.06)	1.36(0.90, 2.07)	1.27(0.83, 1.94)	25/413692	0.95(0.54, 1.67)	0.91(0.52, 1.62)	0.83(0.46, 1.48)
Quartile 3	63/347688	1.50(1.00, 2.25)^a^	1.45(0.96, 2.19)	1.28(0.84, 1.97)	23/413348	0.82(0.46, 1.47)	0.75(0.41, 1.35)	0.59(0.31, 1.11)
Quartile 4	74/348715	1.74(1.17, 2.59)^a^	1.53(1.01, 2.32)^a^	1.26(0.79, 2.00)	26/413908	0.98(0.56, 1.73)	0.78(0.43, 1.43)	0.47(0.22, 0.98)
P‐trend		0.006	0.07	0.43		0.93	0.38	0.03
Continuous per 5‐unit increase		1.11(1.03, 1.19)	1.07(0.99, 1.16)	1.03(0.93, 1.14)		1.03(0.93, 1.14)	0.99(0.88, 1.10)	0.86(0.74, 0.99)

Model 1: Age (*37–49, 50–59, or ≥75 years*) stratified model

Model 2: Additionally adjusted for ethnic (*white, non‐white*), index of multiple deprivation (*fifth*), alcohol consumption (*daily or almost daily, three or four times a week, once or twice a week, one to three times a month, special occasions only, never, or unknown/missing*), smoking status (*never smoker, previous smoker, or current smoker*), physical activity (*low, moderate, or high)*, fruit and vegetable intake (*≥5 portions or <5 portions*), diabetes (*yes or no*), height (*75–159, 160–169,170–179 or 180–209 cm*).

Model 3: Additionally, mutually adjusted for fat free mass and fat mass.

^a^ 0.01 < *p* ≤ 0.05

^b^ 0.001 < *p* ≤ 0.005.

**FIGURE 1:**
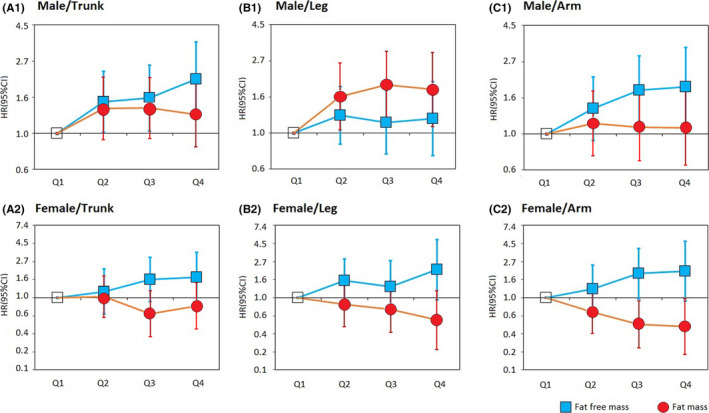
Associations between distribution of fat‐free mass/fat mass and risk of gastric cancer. HR, hazard ratio; CI, confidence interval; Q1, Quartile 1; Q2, Quartile 2; Q3, Quartile 3; Q4, Quartile 4; The analyses were stratified by age (*37–49, 50–59, or ≥75 years*), and additionally adjusted for ethnic (*white, non‐white*), index of multiple deprivation (*fifth*), alcohol consumption (*daily or almost daily, three or four times a week, once or twice a week, one to three times a month, special occasions only, never, or unknown/missing*), smoking status (*never smoker, previous smoker, or current smoker*), physical activity (*low, moderate, or high)*, fruit and vegetable intake (*≥5 portions or <5 portions*), diabetes (*yes or no*), height (*75–159, 160–169,170–179 or 180–209 cm*), NSAIDS use (*yes or no*), and family history of cancer (*yes or no*), and mutually adjusted for fat‐free mass and fat mass.

We further evaluated the risk of gastric cancer according to the distribution of fat‐free mass/fat mass (Figure 1). For both genders, fat‐free mass was likely to associate with an increased risk of gastric cancer, particularly for those distributed in the trunk and arm in males. For fat mass, the associations varied between males and females. Compared with male participants in the lowest quartile of leg body fat mass, those in the second (Adjusted HR 1.66, 95% CI 1.05 to 2.63), third (Adjusted HR 1.95, 95% CI 1.23 to 3.10) and highest quartile (Adjusted HR 1.83, 95% CI 1.09 to 3.05) were associated with increased risk of gastric cancer. We did not observe sufficient evidence of associations between trunk/arm fat mass with gastric cancer in males. For females, those in the third (Adjusted HR 0.48, 95% CI 0.25 to 0.93) and highest quartile (Adjusted HR 0.45, 95% CI 0.21 to 0.98) of arm fat mass was associated with a decreased risk of gastric cancer as compared with those in the lowest quartile.

The associations between traditional anthropometric measures, including BMI and WC, with risk of gastric cancer were presented in Table 3. In male participants, each 5‐unit increase in BMI and WC was associated with a 17% (Adjusted HR 1.17, 95% CI 1.01 to 1.37) and 9% (Adjusted HR 1.09, 95% CI 1.02 to 1.15) increase in the risk of gastric cancer, respectively. We did not observe sufficient evidence of associations between these anthropometric measures with gastric cancer in females.

**TABLE 3:** Risk of gastric cancer according to body mass index and waist circumference

	Males			Females		
	Case/ person‐years	Hazard Ratio (95% Confidence Interval)		Case/ person‐years	Hazard Ratio (95% Confidence Interval)	
		Model 1	Model 2		Model 1	Model 2
Body mass index Quartiles						
Quartile 1	38/349079	1.00(Reference)	1.00(Reference)	24/414594	1.00(Reference)	1.00(Reference)
Quartile 2	56/348681	1.41(0.93, 2.13)	1.42(0.87, 2.32)	23/413926	0.85(0.48, 1.51)	0.98(0.56, 1.72)
Quartile 3	69/348241	1.73(1.16, 2.57)^b^	1.91(1.19, 3.04)^b^	22/413857	0.75(0.42, 1.35)	0.91(0.51, 1.63)
Quartile 4	66/347723	1.69(1.13, 2.51)^b^	1.74(1.07, 2.84)^a^	28/412772	1.01(0.59, 1.75)	0.90(0.51, 1.61)
P‐trend		0.01	0.09		0.87	0.51
Continuous per 5‐unit increase Waist circumference Quartiles		1.25(1.08, 1.44)^a^	1.17(1.01, 1.37)^a^		1.12(0.93, 1.35)	1.03(0.84, 1.26)
Quartile 1	27/313565	1.00(Reference)	1.00(Reference)	20/367629	1.00(Reference)	1.00(Reference)
Quartile 2	52/364109	1.52(0.96, 2.42)	2.82(0.67, 11.78)	24/440833	0.87(0.48, 1.58)	0.99(0.6, 1.62)
Quartile 3	62/337724	1.85(1.18, 2.91)^b^	4.09(1.00, 16.70)^a^	25/415978	0.88(0.49, 1.59)	0.50(0.25, 1.04)
Quartile 4	88/379305	2.27(1.47, 3.50)^c^	4.65(1.14, 18.91)^b^	28/430853	0.95(0.53, 1.69)	1.36(0.75, 2.45)
P‐trend		<0.001	<0.001		0.96	0.37
Continuous per 5‐unit increase		1.11(1.05, 1.17)^c^	1.09(1.02, 1.15)^b^		1.05(0.97, 1.13)	1.01(0.93, 1.10)

Model 1: Age (*37–49, 50–59, or ≥75 years*) stratified model;

Model 2: Additionally adjusted for ethnic (*white, non‐white*), index of multiple deprivation (*fifth*), alcohol consumption (*daily or almost daily, three or four times a week, once or twice a week, one to three times a month, special occasions only, never, or unknown/missing*), smoking status (*never smoker, previous smoker, or current smoker*), physical activity (*low, moderate, or high)*, fruit and vegetable intake (≥5 portions or <5 portions), diabetes (*yes or no*), height (*75–159, 160–169,170–179 or 180–209 cm*), NSAIDS use (*yes or no*), and family history of cancer (*yes or no*).

^a^ 0.01 < *p* ≤ 0.05

^b^ 0.001 < *p* ≤ 0.005

^c^ *p* ≤ 0.001.

**FIGURE 2:**
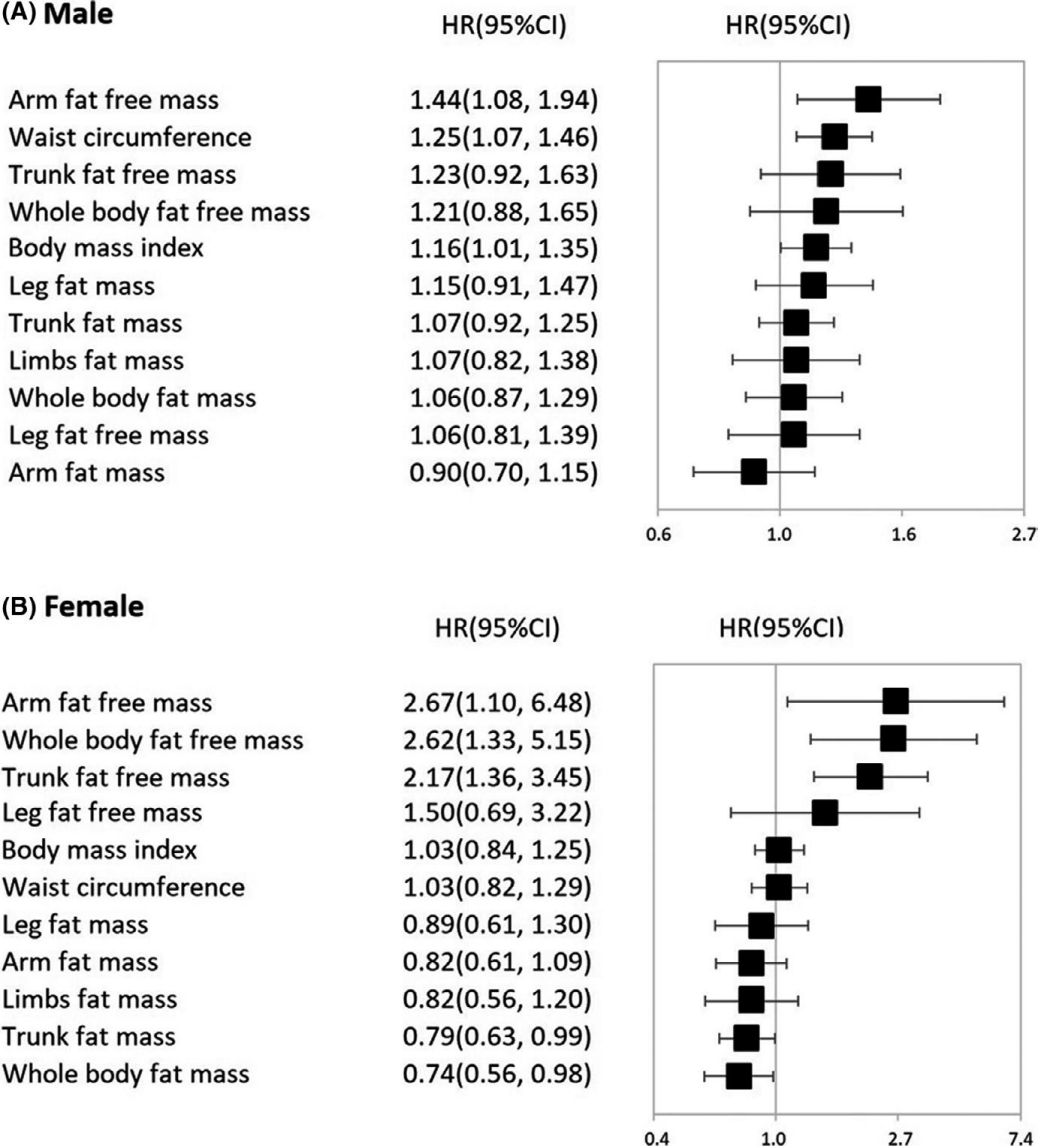
Hazard ratio per SD increase in gastric cancer risk across various body composition and anthropometry measures. The analyses were stratified by age (*37–49, 50–59, or ≥75 years*), and additionally adjusted for ethnicity (*white, non‐white*), index of multiple deprivation (*fifth*), alcohol consumption (*daily or almost daily, three or four times a week, once or twice a week, one to three times a month, special occasions only, never, or unknown/missing*), smoking status (*never smoker, previous smoker, or current smoker*), physical activity (*low, moderate, or high)*, fruit and vegetable intake (*≥5 portions or <5 portions*), diabetes (*yes or no*), height (*75–159, 160–169, 170–179 or 180–209 cm*), NSAIDS use (*yes or no*), and family history of cancer (*yes or no*). For fat‐free mass and fat mass, mutually adjusted was applied.

In the evaluation of gastric cancer risk across various body composition and anthropometry measures, arm fat‐free mass showed the highest HR per SD increase in gastric cancer risk in males (Adjusted HR 1.44, 95% CI 1.08 to 1.94), followed by WC (Adjusted HR 1.25, 95% CI 1.07 to 1.46) (Figure 2). For females, arm fat‐free mass also showed the highest HR per SD increase in gastric cancer risk (Adjusted HR 2.67, 95% CI 1.10 to 6.48), followed by whole body fat‐free mass (Adjusted HR 2.62, 95% CI 1.10 to 5.15) and trunk fat‐free mass (Adjusted HR 2.17, 95% CI 1.36 to 3.45).

Table 4 presents the risk of GCA and NGCA according to various body composition and anthropometry measures. We did not perform the analysis by gender as the number of cases was small. For GCA, BMI (HR per 5‐unit increase 1.19, 95% CI 0.98 to 1.44) and WC (HR per 5‐unit increase 1.09, 95% CI 1.02 to 1.18) were associated with increased risk. For NGCA, we observed that whole body fat free mass (HR per 5‐unit increase 1.23, 95% CI 1.05 to 1.45) was associated with increased risk. We did not find sufficient evidence of associations between whole body fat mass, BMI, and WC with risk of NGCA.

**TABLE 4:** Associations between anthropometric measurements and risk of gastric cardia adenocarcinoma and non‐gastric cardia adenocarcinoma.

	Gastric cardia adenocarcinoma		Non‐gastric cardia adenocarcinoma	
	Case/person‐years	HR (95% CI)	Case/person‐years	HR (95% CI)
Whole body fat‐free mass Quartiles				
Quartile 1	11/755771	1.00(Reference)	30/755771	1.00(Reference)
Quartile 2	14/772667	0.91(0.39, 2.16)	38/772667	1.74(1.02, 2.98)^a^
Quartile 3	46/759315	1.02(0.33, 3.14)	55/759315	2.04(0.94, 4.46)
Quartile 4	64/762598	1.23(0.35, 4.27)	68/762598	3.31(1.32, 8.28)^b^
P value for trend		0.46		0.01
Continuous per 5‐unit increase Whole body fat mass Quartiles		1.06(0.88, 1.27)		1.23(1.05, 1.45)^a^
Quartile 1	28/751992	1.00(Reference)	40/751992	1.00(Reference)
Quartile 2	31/771785	1.09(0.65, 1.83)	56/771785	1.17(0.77, 1.77)
Quartile 3	41/758246	1.48(0.88, 2.47)	49/758246	0.90(0.58, 1.41)
Quartile 4	35/768329	1.47(0.81, 2.66)	46/768329	0.70(0.42, 1.17)
P value for trend		0.15		0.12
Continuous per 5‐unit increase Body mass index Quartiles		1.06(0.94, 1.21)		0.92(0.83, 1.03)
Quartile 1	19/764601	1.00(Reference)	31/764601	1.00(Reference)
Quartile 2	26/762934	1.01(0.56, 1.83)	49/762934	1.27(0.81, 2.00)
Quartile 3	46/761072	1.58(0.92, 2.72)	58/761072	1.33(0.85, 2.08)
Quartile 4	44/760267	1.62(0.92, 2.84)	53/760267	1.14(0.71, 1.81)
P value for trend		0.04		0.79
Continuous per 5‐unit increase Waist circumference Quartiles		1.19(0.98, 1.44)		1.07(0.92, 1.26)
Quartile 1	10/699360	1.00(Reference)	25/699360	1.00(Reference)
Quartile 2	21/808173	0.97(0.44, 2.12)	41/808173	1.04(0.62, 1.74)
Quartile 3	34/762488	1.16(0.54, 2.50)	54/762488	1.16(0.69, 1.95)
Quartile 4	70/779975	1.96(0.92, 4.15)	71/779975	1.23(0.72, 2.10)
P value for trend		0.003		0.36
Continuous per 5‐unit increase		1.09(1.02, 1.18)^a^		1.04(0.97, 1.1)

HR, hazard ratio; CI, confidence interval.

The analyses were stratified by age (*37–49, 50–59, or ≥75 years*) and additionally adjusted for ethnic (*white, non‐white*), index of multiple deprivation (*fifth*), alcohol consumption (*daily or almost daily, three or four times a week, once or twice a week, one to three times a month, special occasions only, never, or unknown/missing*), smoking status (*never smoker, previous smoker, or current smoker*), physical activity (*low, moderate, or high*), fruit and vegetable intake (*≥5 portions or <5 portions*), diabetes (*yes or no*), height (*75–159, 160–169,170–179 or 180–209 cm*), NSAIDS use (*yes or no*), and family history of cancer (*yes or no*). For whole body fat‐free mass and whole body fat mass, mutually adjusted was applied.

^a^ 0.01 < *p* ≤ 0.05

^b^ 0.001 < *p* ≤ 0.005.

*Electronic supplementary material* Table S1 presents the sensitivity analyses. The primary results were stable in the analyses by limiting the participants in people with a follow‐up time over 2 years, additionally adjusted for gastro‐oesophageal reflux/gastric reflux, gastric/stomach ulcers, gastritis/gastric erosions, and PPI use, additionally adjusted for HRT and OCT in females, excluded participants with self‐reported cancer diagnosed by doctor, additionally adjusted red meat consumption.

## 4 DISCUSSION

In this prospective cohort of over 0.46 million participants, we observed that fat‐free mass, particularly those distributed in arm and trunk in females, was associated with an increased risk of gastric cancer. While whole body fat mass and arm fat mass were associated with a decreased risk of gastric cancer in females. For both genders, arm fat‐free mass was likely to be the strongest predictor of gastric cancer risk. Traditional anthropometric measures, including BMI and WC, were associated with increased risk of gastric cancer in males, but not in females. Collectively, these findings indicated that fat‐free mass and fat mass may play a different role in gastric cancer development in males and females.

Epidemiological studies evaluating the associations between body composition and gastric cancer risk remain inadequate. In 2006, a prospective analysis of 41,295 participants from the Melbourne Collaborative Cohort Study^8^ suggested that fat‐free mass (HR per 10 kg 2.06, 95% CI 1.15 to 3.69) was associated with increased risk of lower oesophagus/gastric cardia cancer. However, there was no sufficient evidence that fat‐free mass (HR per 10 kg 1.26, 95% CI 0.80 to 1.96) and fat mass (HR per 10 kg 1.01, 95% CI 0.76 to 1.36) were associated with increased risk of NGCA. These results were not in agreement with ours, which showed that fat‐free mass was associated with increased risk of NGCA but not with risk of GCA. The inconsistencies may be due to the difference in covariate adjustment. The previous study only adjusted for gender, country of birth, education, and physical activity, while adjustment for other important confounders, such as smoking, diet, and family history of cancer, and mutual adjustment for fat‐free mass and fat mass, were not carried out. Our analysis and previous studies^11^, ^12^, ^13^ showed that adjustment for these factors could lead to a major change in the estimated effect. It is very likely that the results would change if these adjustments were done. Similar to our results, this study also showed that BMI and WC tended to associate with increased risk of GCA.^8^Such results have also been shown in previous meta‐anlayses.^14^, ^15^

This study indicated that fat‐free mass, rather than fat mass, was associated with increased risk of gastric cancer. Similar findings have also shown in previous studies evaluating body composition and risk of lower oesophagus/gastric cardia,^8^ breast cancer,^12^, ^16^ prostate cancer,^17^ rectal cancer,^18^ and lung cancer.^11^ The exact mechanisms of fat‐free mass on cancer risk remain unclear and may vary among different cancer sites. A potential explanation is that fat‐free mass is associated with nutritional factors, such as red meat consumption, which is linked to risk of gastric cancer.^19^ In addition, fat‐free mass may be associated with sex hormone,^20^ insulin resistance,^21^, ^22^ which were likely to be linked with gastric cancer.^23^, ^24^, ^25^ Regarding fat mass, we observed a protective effect in females. A possible explanation is that adipose tissue is a major source of estrogen,^26^ while longer exposure to estrogenic effects may decrease risk of gastric cancer.^24^

Our study found body composition, particularly fat mass, had a different effect on gastric cancer risk between males and females. A gender difference have also been shown on the associations between body compositions and risk of colon cancer^13^, ^27^ and lung cancer.^11^ To the best of our knowledge, no previous study has evaluated the gender‐specific associations between body composition and gastric cancer. Our analysis for BMI and WC also indicated that there is a gender difference in gastric cancer risk. A significantly increased risk was shown only in males. The results were in agreement with a previous meta‐analysis of ten studies, which showed that overweight (Odd Ratio [OR] 1.07, 95% CI 1.01–1.03) and obese (OR 1.12, 95% CI 1.00–1.24) males were associated with an increased risk of gastric cancer, but no sufficient evidence of associations was shown in females (overweight: OR 0.99, 95% CI 0.89–1.11; obesity: OR 1.04, 95% CI 0.93–1.16).^28^ Similar findings were also shown in another meta‐analysis.^15^ To the contrary, in a recent meta‐analysis of Asian adults, overweight and obesity showed a protective effect (Relative Risk [RR] 0.79, 95% CI 0.89–1.11) in males while there was no association in females (RR 1.08, 95% CI 0.72–1.63) with gastric cancer risk. The inconsistency among these studies might be due the differences in race, and definitions of obesity/overweight.

### 4.1 Strength and limitations

To the best of our knowledge, the present study is currently the largest epidemiological study evaluating body composition and gastric cancer risk. The analysis was based on a well‐established nationwide cohort of over 0.45 million participants, with detailed measurements of body composition and a wide range of known and putative gastric cancer risk factors, allowing us to adequately control potential confounding factors. For the first time, we evaluated the gender‐specific effect of body composition on gastric cancer risk. In addition, we applied a range of methodological approaches to evaluate body composition effects, including the assessment of non‐linearity and effect modification. Lastly, a wide range of robust sensitivity analyses further strengthened our confidence in the results.

This study has its limitations. First, as an observational study, we cannot eliminate residual confounding effect and confirm the causal relationship. Second, due to a low gastric cancer incidence in European countries, the number of cases was low particularly for the analyses of GCA and NGCA, so some estimated effects were imprecise. Third, assessment of body composition by bioelectrical impedance analysis may be influenced by factors such as the environment, ethnicity, phase of menstrual cycle, and underlying medical conditions.^29^ However, the high correlations between bio‐impedance measures and the DXA‐derived, which is considered as gold standard, indicated that bio‐impedance is reliable. Last, as most participants in the UK Biobank were of European ancestry, the generalizability of the study findings to other ethnicities remained unclear.

### 4.2 Implication

Overall, this large‐scale prospective study suggested that fat‐free mass tended to associate with increased risk of gastric cancer in both genders, while fat mass was associated with reduced gastric cancer risk in females. For both genders, arm fat‐free mass was likely to be the strongest predictor of gastric cancer risk. In clinical practice, our findings provided evidence for individualized weight management for the prevention of gastric cancer. For people with high arm fat‐free mass, and other gastric cancer risk factors, regular cancer screening is recommended. Interventions for controlling excessive fat free mass may have benefits in reducing gastric cancer risk, although more research is still needed to confirm the causal relationship. For future research, this study suggested that fat‐free mass and fat mass may play a different role in gastric cancer development. Traditional anthropometric measures, including BMI and WC, were insufficient to precisely predict gastric cancer risk. Further research is warranted to confirm the causality and to investigate the underline mechanism of the gender‐specific effects of fat‐free mass/fat mass on gastric cancer.

## CONFLICT OF INTEREST

None declared.

## Supporting information

Supplementary Material

## Data Availability

The data that support the findings of this study are openly available in UK Biobank website (http://www.ukbiobank.ac.uk/).
