# Supplementary material for: Body composition and risk of gastric cancer: A population‐based prospective cohort study
Source: Cancer Med. 2021 Feb 23;10(6):2164–74. doi: 10.1002/cam4.3808 (PMC7957174; doi:10.1002/cam4.3808)
Supplement: Supplementary file 1 — Supplementary Material [file CAM4-10-2164-s001.docx]

**Electronic supplementary material**


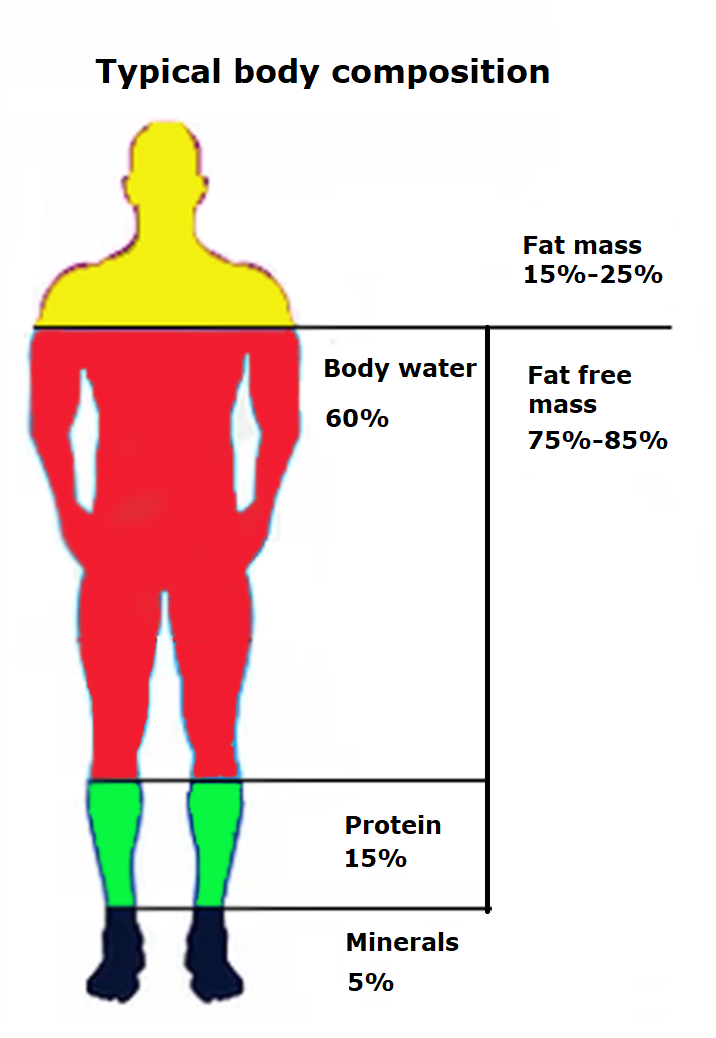


**Figure S1:** Typical body composition

465 292 Participants in the final dataset

502 527 Participants in the UK Biobank

491503 Participants remaining

26 211 Participants with any cancer diagnosis prior to baseline

11 024 Participants without complete information on body composition

**Figure S2:** Flow chart of participant inclusion and exclusion


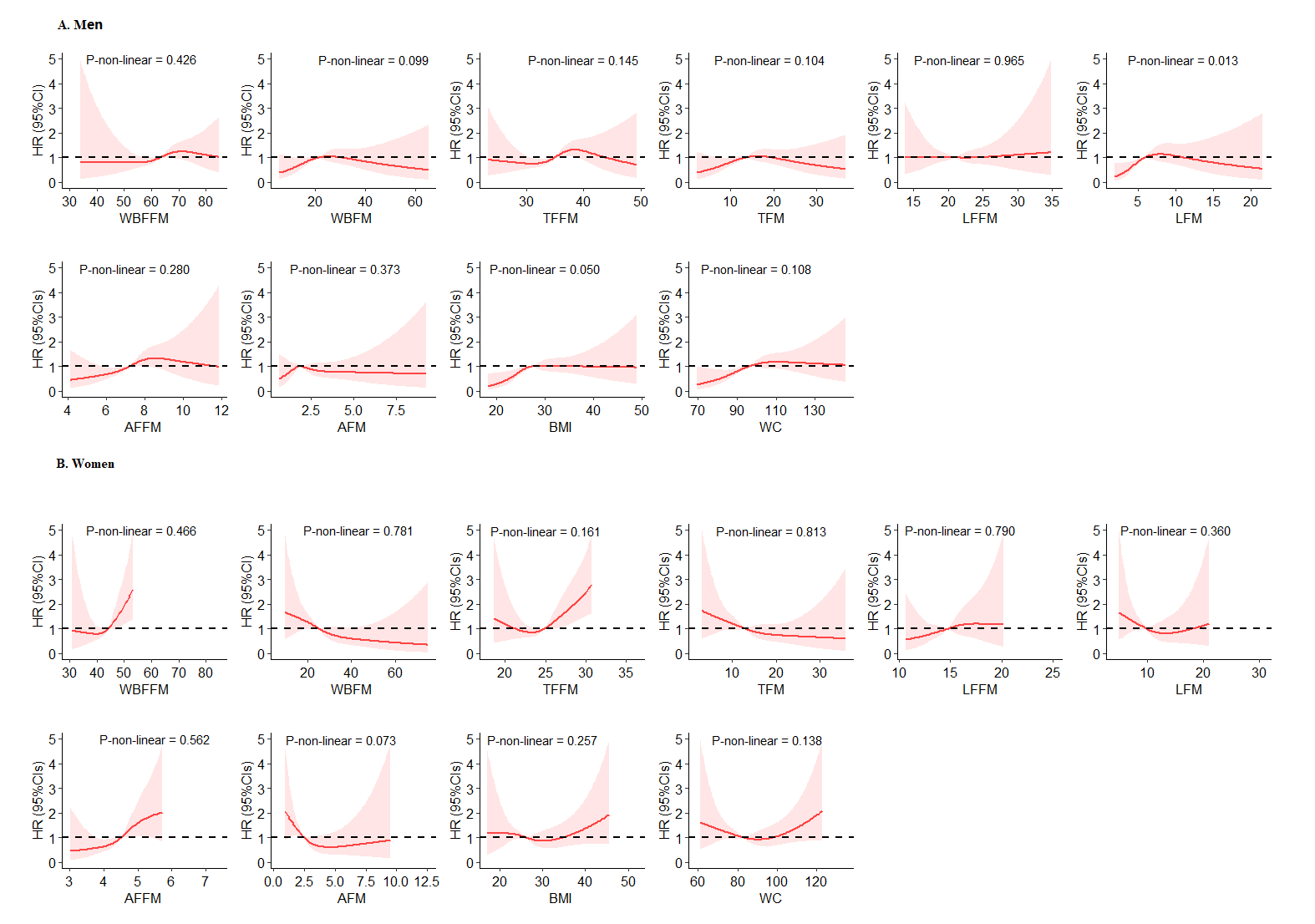


**Figure S3:** Associations of body composition, BMI, WC with gastric cancer risk, allowing for non-linear effects

Abbreviations: HR, hazard ratio; WBFFM: whole body fat free mass; WBFM: whole body fat mass; TFFM: trunk fat free mass; TFM: trunk fat mass; LFFM: leg fat free mass; LFM: leg fat mass; AFFM: arm fat free mass; AFM: arm fat mass; BMI: body fat mass index; WC: waist circumference.

The models were stratified by age, and adjusted for ethnic, index of multiple deprivation, alcohol consumption, smoking status, physical activity, fruit and vegetable intake, diabetes, height, NSAIDS use, and family history of cancer. For body composition, the analyses were additionally mutually adjusted for fat free mass and fat mass.

**Table S1.** Associations between whole body fat free mass/whole body fat mass and risk of gastric cancer: sensitivity analyses

|  | **Males** | |  | **Females** | |
| --- | --- | --- | --- | --- | --- |
|  | **No of cases/ Person-years** | **HR(95%CI)** |  | **No of cases/ Person-years** | **HR(95%CI)** |
| **Limiting the participants in people with follow up above 2 years** | | | | | |
| Whole body fat free mass |  |  |  |  |  |
| Q1 | 38/344782 | 1.00(Reference) |  | 17/409343 | 1.00(Reference) |
| Q2 | 46/344052 | 1.42(0.89, 2.26) |  | 20/415638 | 1.57(0.79, 3.10) |
| Q3 | 37/352927 | 1.25(0.73, 2.13) |  | 14/408815 | 1.43(0.64, 3.17) |
| Q4 | 48/348743 | 1.97(1.09, 3.57) |  | 23/418026 | 3.07(1.29, 7.31) |
| *P value for trend* |  | 0.197 |  |  | 0.017 |
| Whole body fat mass |  |  |  |  |  |
| Q1 | 25/342236 | 1.00(Reference) |  | 16/413807 | 1.00(Reference) |
| Q2 | 48/354171 | 1.61(0.98, 2.62) |  | 23/412792 | 1.09(0.57, 2.10) |
| Q3 | 42/346601 | 1.27(0.76, 2.14) |  | 16/412364 | 0.59(0.28, 1.24) |
| Q4 | 54/347496 | 1.33(0.76, 2.32) |  | 19/412859 | 0.47(0.2, 1.11) |
| *P value for trend* |  | 0.351 |  |  | 0.033 |
| **Additionally adjusted for gastro-oesophageal reflux/gastric reflux, gastric/stomach ulcers, gastritis/gastric erosions and PPI use** | | | | | |
| Whole body fat free mass |  |  |  |  |  |
| Q1 | 46/346049 | 1.00(Reference) |  | 23/410209 | 1.00(Reference) |
| Q2 | 67/345128 | 1.65(1.10, 2.47) |  | 22/416519 | 1.28(0.69, 2.38) |
| Q3 | 58/353914 | 1.50(0.95, 2.37) |  | 24/409769 | 1.76(0.91, 3.42) |
| Q4 | 58/349725 | 1.72(1.01, 2.92) |  | 28/419038 | 2.53(1.17, 5.44) |
| P value for trend |  | 0.215 |  |  | 0.004 |
| Whole body fat mass |  |  |  |  |  |
| Q1 | 37/349876 |  |  | 23/414586 |  |
| Q2 | 55/348536 | 1.27(0.83, 1.93) |  | 23/414586 | 0.81(0.45, 1.45) |
| Q3 | 63/347688 | 1.27(0.83, 1.95) |  | 25/413692 | 0.56(0.29, 1.05) |
| Q4 | 74/348715 | 1.24(0.78, 1.98) |  | 23/413348 | 0.43(0.20, 0.90) |
| P value for trend |  | 0.376 |  |  | 0.024 |
| **Additionally adjusted for red meat intake** | | | | | |
| Whole body fat free mass |  |  |  |  |  |
| Q1 | 46/346049 | 1.00(Reference) |  | 23/410209 | 1.00(Reference) |
| Q2 | 67/345128 | 1.64(1.09, 2.46) |  | 22/416519 | 1.27(0.68, 2.35) |
| Q3 | 58/353914 | 1.48(0.94, 2.34) |  | 24/409769 | 1.74(0.9, 3.38) |
| Q4 | 58/349725 | 1.69(1, 2.87) |  | 28/419038 | 2.49(1.15, 5.36) |
| P value for trend |  | 0.123 |  |  | 0.015 |
| Whole body fat mass |  |  |  |  |  |
| Q1 | 37/349876 | 1.00(Reference) |  | 23/414586 | 1.00(Reference) |
| Q2 | 55/348536 | 1.26(0.83, 1.93) |  | 25/413692 | 0.84(0.47, 1.51) |
| Q3 | 63/347688 | 1.27(0.83, 1.95) |  | 23/413348 | 0.59(0.31, 1.12) |
| Q4 | 74/348715 | 1.24(0.78, 1.97) |  | 26/413908 | 0.47(0.23, 0.99) |
| P value for trend |  | 0.472 |  |  | 0.036 |
| **Limiting the participants without self-reported cancer diagnosed** | | | | | |
| Whole body fat free mass |  |  |  |  |  |
| Q1 | 45/338323 | 1.00(Reference) |  | 21/395920 | 1.00(Reference) |
| Q2 | 65/337755 | 1.62(1.07, 2.45) |  | 22/402668 | 1.38(0.73, 2.6) |
| Q3 | 57/346320 | 1.5(0.95, 2.38) |  | 23/395913 | 1.8(0.91, 3.57) |
| Q4 | 57/342652 | 1.73(1.01, 2.95) |  | 28/405523 | 2.64(1.21, 5.78) |
| P value for trend |  | 0.231 |  |  | 0.004 |
| Whole body fat mass |  |  |  |  |  |
| Q1 | 35/336231 | 1.00(Reference) |  | 22/401308 | 1.00(Reference) |
| Q2 | 55/347950 | 1.3(0.84, 1.99) |  | 24/399911 | 0.82(0.45, 1.48) |
| Q3 | 62/340087 | 1.31(0.85, 2.03) |  | 22/398758 | 0.57(0.3, 1.1) |
| Q4 | 72/340783 | 1.28(0.8, 2.06) |  | 26/400045 | 0.47(0.22, 0.99) |
| P value for trend |  | 0.386 |  |  | 0.044 |
| **Additionally adjusted for HRT and OCT in females** | | |  |  |  |
| Whole body fat free mass |  |  |  |  |  |
| Q1 | - | - |  | 23/410209 | 1.00(Reference) |
| Q2 | - | - |  | 22/416519 | 1.77(0.91, 3.44) |
| Q3 | - | - |  | 24/409769 | 2.55(1.18, 5.50) |
| Q4 | - | - |  | 28/419038 | 1.07(1.02, 1.11) |
| *P value for trend* |  |  |  |  | 0.004 |
| Whole body fat mass |  |  |  |  |  |
| Q1 | - | - |  | 23/414586 | 1.00(Reference) |
| Q2 | - | - |  | 25/413692 | 0.55(0.29, 1.04) |
| Q3 | - | - |  | 23/413348 | 0.42(0.20, 0.88) |
| Q4 | - | - |  | 26/413908 | 0.96(0.93, 0.99) |
| *P value for trend* |  |  |  |  | 0.002 |

The analyses were based on the fully-adjusted model (see foot note in table 2 in the main text)
